# Bronchial Fibroepithelial Polyp With Severe Hemoptysis as First Manifestation: A Case Report

**DOI:** 10.7759/cureus.10261

**Published:** 2020-09-05

**Authors:** Vasiliki E Georgakopoulou, Eftychia Kourtelesi, Dimitrios Mermigkis, Nikolaos Trakas, Xanthi Tsiafaki

**Affiliations:** 1 Pulmonology Department, Laiko General Hospital, Athens, GRC; 2 1st Pulmonology Department, Sismanogleio Hospital, Athens, GRC; 3 Intensive Care Unit, Sismanogleio Hospital, Athens, GRC; 4 Biochemistry Department, Sismanogleio Hospital, Athens, GRC

**Keywords:** fibroepithelial polypod, hemoptysis

## Abstract

Fibroepithelial polyps are uncommon benign tumors that mostly occur in skin, oral cavity and genitourinary tract. These lesions have common morphological features with other mesenchymal tumors such as angiomyofibroblastoma, aggressive angiomyxoma, and cellular angiofibroma and present with numerous histological appearances. Benign endobronchial tumors are rare. These neoplasms have a slow growth and usually are related to bronchial obstruction. Only a few cases of bronchial fibroepithelial polyps have been reported. Bronchial fibroepithelial polyps might present with airway stenosis resulting in atelectasis and bronchiectasis and the most frequent manifestations are recurrent infection, refractory asthma, dyspnea and hemoptysis. Chronic inflammation is associated with the pathogenesis of fibroepithelial polyps. Treatment varies according to mainly to the size and symptoms. Small lesions presenting with few symptoms can be treated with corticosteroids and antibiotics while invasive techniques including bronchoscopic resection of the polyp or lobectomy are used for larger lesions. We report a case of a bronchial fibroepithelial polyp with severe hemoptysis as the first manifestation. Physicians should always suspect these lesions in the differential diagnosis of hemoptysis and with initial right diagnosis surgical procedures can be avoided.

## Introduction

Fibroepithelial polyps represent rare benign tumors that are most frequently present in skin, oral cavity and genitourinary tract [1]. These lesions have overlapping morphological characteristics with other mesenchymal tumors such as angiomyofibroblastoma, aggressive angiomyxoma, and cellular angiofibroma and have a wide range of histological appearances [2].

Benign endobronchial tumors are uncommon. These neoplasms present with slow growth and usual manifestation is related to bronchial obstruction [3]. Benign bronchial polypoid lesions are uncommon and include non-neoplastic conditions such as vascular malformations and granulation tissue, mesenchymal tumors such as hamartomas, lipomas and inflammatory myofibroblastic tumours, and epithelial tumors such as papillomas and salivary gland adenomas [4]. Only a few cases of bronchial fibroepithelial polyps have been described [4].

Bronchial fibroepithelial polyps might present with airway stenosis resulting in recurrent respiratory infections, atelectasis and bronchiectasis. The most common reasons for admission are recurrent infection, refractory asthma, dyspnea and hemoptysis [5]. Chronic inflammation is considered as an important factor in the pathogenesis of fibroepithelial polyps. Smoking, chronic inflammation associated with asthma and chronic obstructive pulmonary disease, infections, foreign body aspiration and prolonged mechanical ventilation are possible underlying causes [5]. Treatment varies according to the size, symptoms and hardness of the lesion. Small lesions presenting with minimal symptoms can be treated with corticosteroids and antibiotics while invasive techniques include bronchoscopic resection of the polyp or lobectomy. Bronchoscopic treatment is essential for large lesions and surgical treatment is needed when performing bronchoscopic resection is difficult or the histological features are controversial [6]. We report a case of a bronchial fibroepithelial polyp with severe hemoptysis as the first manifestation.

## Case presentation

A 66-year-old female, non-smoker, patient was admitted to our emergency department for severe hemoptysis over the last two days. She had a history of dyslipidemia and atrial fibrillation ablation in the past.

A few months ago, she was hospitalised with hemoptysis and during this hospitalisation, she underwent computerized tomography (CT) of the chest examining the bronchial arterial anatomy which did not reveal dilated bronchial arteries. CT showed bronchiectasis in the right lower lobe and thickening of the posterior wall of the left main bronchus. The patient underwent bronchoscopy with flexible bronchoscope and active bleeding from lower left lobe was found. Argon plasma coagulation was performed and mucosal biopsies and bronchial washings for cytological and microbiological examination were obtained, without abnormal findings.

On admission, clinical examination revealed crackles on auscultation at both lung bases. Blood pressure was 130/95 mmHg, heart rate was 90 beats per minute, oxygen saturation was 93% on room air and body temperature 36.8°C, without abnormal findings on electrocardiography. Chest X-ray showed a consolidation in the right lower lobe.

**Figure 1:**
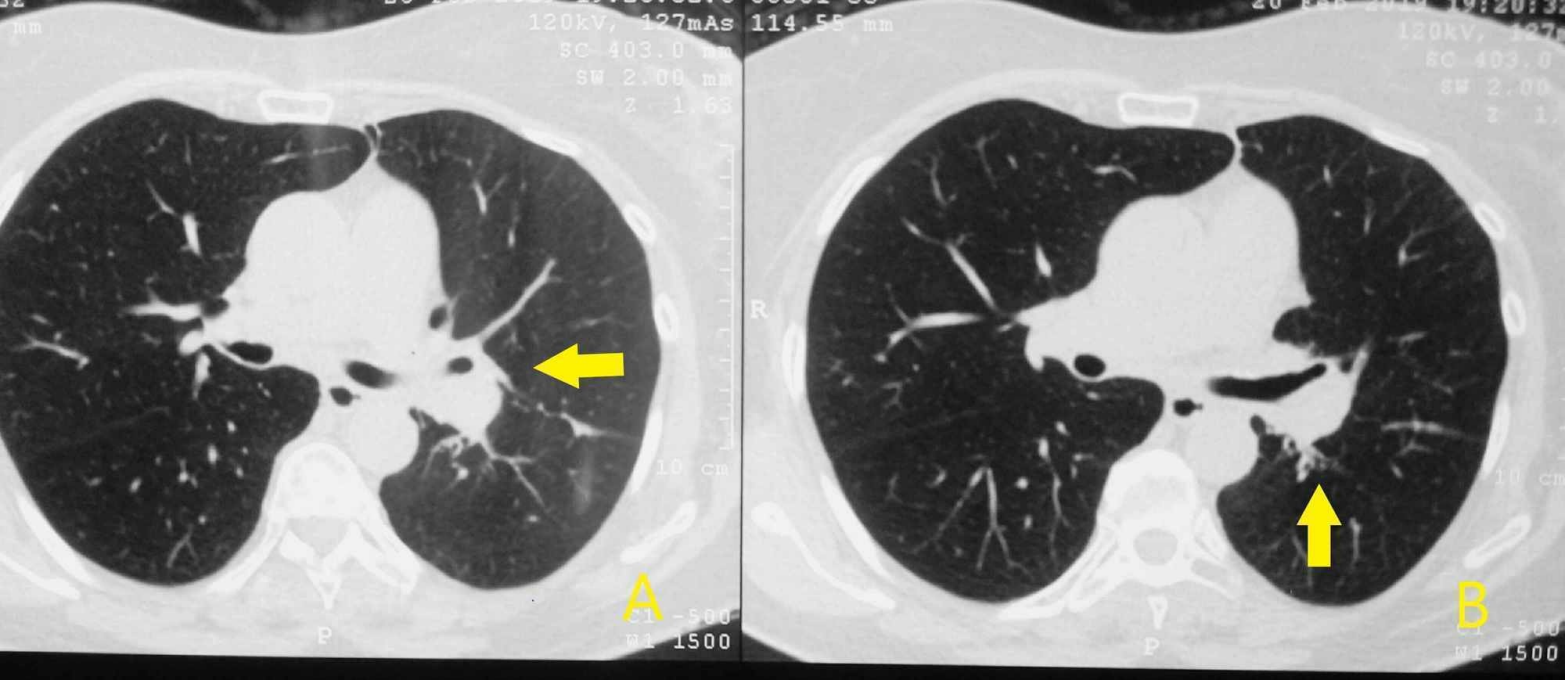
Computerized tomography of the chest A, B: Arrows show bronchial wall thickening of the left main bronchus

**Figure 2:**
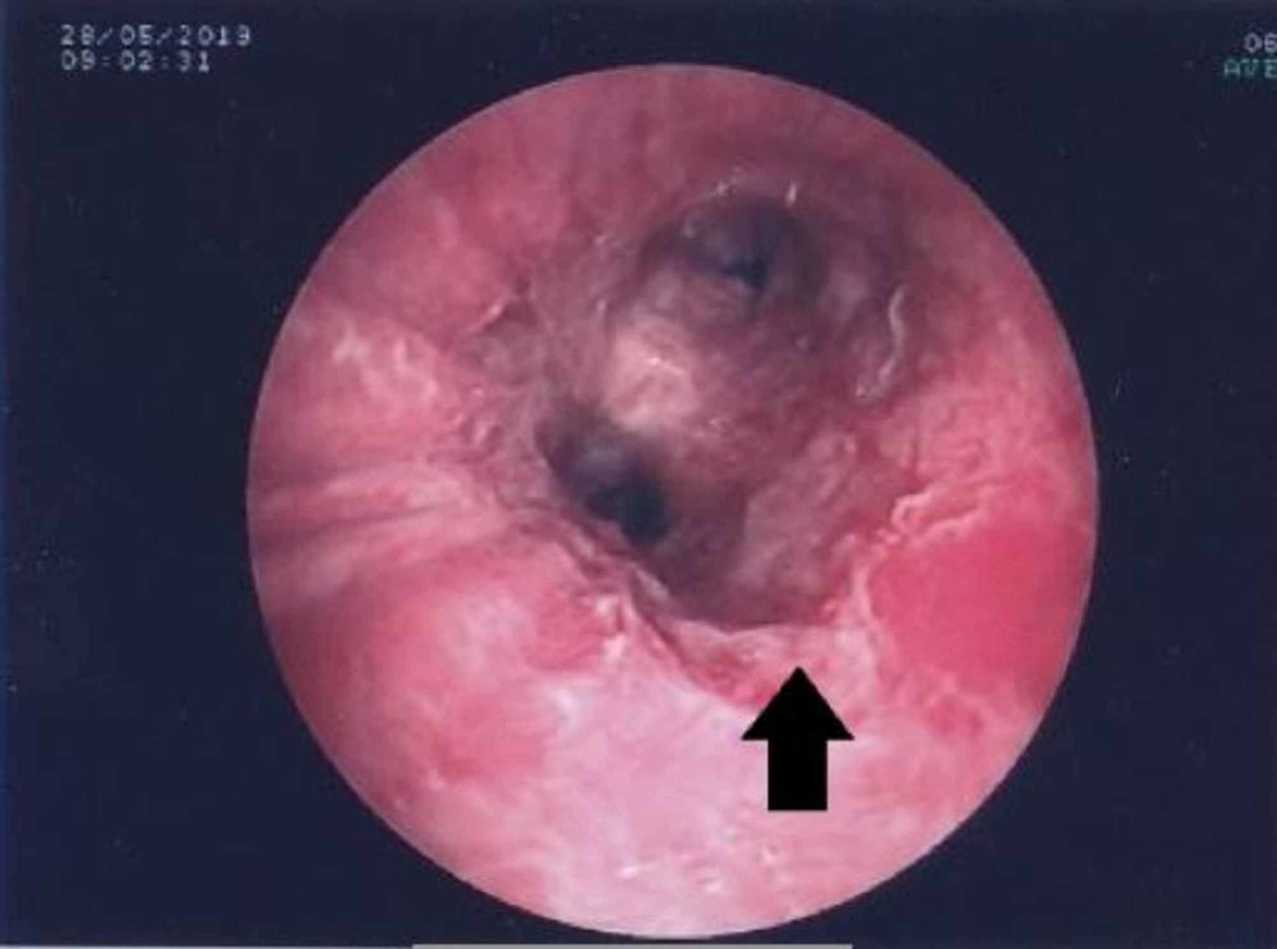
Entrance of left main bronchus Mucosal invasion of the left main bronchus

Laboratory findings, including coagulation tests, were normal. The patient underwent CT of the chest in our hospital, that showed bronchiectasis and ground-glass opacities in the lower lobes and bronchial wall thickening of the left main bronchus, which has already been reported in previous imaging (Figure 1). The patient underwent a new bronchoscopy with fiberoptic bronchoscope which revealed mucosal invasion of the left main bronchus with increased bleeding tendency (Figure 2). Biopsies from affected bronchial mucosa were obtained. Histological examination of these biopsy specimens revealed the presence of fibroepithelial polyp with angiectasis. The patient received conservative therapy for hemoptysis, without other intervention.

## Discussion

This is a rare case of the bronchial fibroepithelial polyp. Fibroepithelial polyps were reported for the first time in 1938 when Pollak, Cohen, and Gnassi in a review of the literature for benign bronchial tumors described 27 cases detected at autopsy and 77 encountered during the life of which flbroepithelial polyps were recognised in 11 living patients. [7]. In 1960, Rowlands reported a 38-year-old man with sharp right anterior chest pain, cough, productive of white-yellow to red-streaked sputum and shortness of breath with progressive intensity and bronchoscopy revealed a multinodular white mass below the level of the right upper lobe bronchial orifice. Histological examination of biopsy specimen showed a fibroepithelial polyp [7].

Dinçer et al. described a case of a 55-year-old male patient with recurrent pulmonary infections for 40 years and a chest CT revealed obstruction of the main bronchus at the level of carina. In bronchoscopy, a mobile polypoid pinkish lesion was observed, the patient underwent bilobectomy and the histological examination revealed an endobronchial fibroepithelial polyp [8]. Strachan et al. reported a 63-year-old man who was referred to the pulmonary department for an abnormal CT revealing a localized lesion within the right upper lobe bronchus, with mild right upper lobe bronchiectasis. A flexible bronchoscopy revealed a pedunculated lesion in the right upper lb bronchus and a lobectomy was performed. The pathology features were those of a benign fibroepithelial polyp [9].

Ushiki et al. reported a case of a 75-year-old female with a productive cough, a solid lobulated nodule in the right main bronchus and subtotal atelectasis of the right lower lobe in CT and bronchoscopy with biopsy confirmed the diagnosis of a bronchial fibroepithelial polyp [6]. Wartmann et al. described a case of a 32-year-old man who was admitted with an indeterminate obstructing lung lesion and thoracotomy revealed a benign fibroepithelial polyp of the bronchus [10]. The authors, in this case, underlined the need for preoperative right diagnosis in order to prevent thoracotomy.

Maskey et al. reported a case of a 39-year-old man, presented with dyspnea, with a fibroepithelial polyp arising from the right upper lobe bronchus and extending into the main stem bronchus, which was successfully removed using an electrocautery snare [11]. Leiro-Fernández et al. described a 77-year-old man diagnosed with a fibroepithelial polyp located at the bifurcation of the left main bronchus that was completely removed using flexible bronchoscope [12]. Melo et al. reported the case of a 45-year-old man, active smoker, admitted with fever, purulent sputum and pleuritic chest pain, progressive dyspnea on exertion, orthopnea and cough with mucoid sputum and she was diagnosed with a fibroepithelial polyp on the right upper lobe carina, totally obstructing the right main bronchus [13]. Labarca et al. reported a 79- year-old male smoker, who was referred with a three-month history of dry cough and a chest CT showed a 6mm polyp lesion in his trachea. Argon plasma coagulation was used to completely resect and treat the lesion and pathological analysis revealed a fibroepithelial polyp [14].

Some authors have described cases of bronchial fibroepithelial polyps underlying the role of different specific bronchoscopic techniques in these cases. Saito et al. described a 65-year-old man, who was diagnosed with a bronchial fibroepithelial polyp of the right basal bronchus and underwent bronchoscopic examination, with a videobronchoscope that incorporates two devices for white-light (WL) mode and auto-fluorescence imaging (AFI). Bronchoscopy under WL mode revealed a rounded, whitish, smooth, and glistening polypoid lesion with a lobulated surface and on AFI, the lesion appeared magenta colour (positivity) [15]. The authors of this case concluded that bronchial fibroepithelial polyps may display AFI positivity and the right diagnosis should not be confused by this positivity [15]. Desai and Bernstein described a 58-year-old male with an endobronchial lesion in the left lower lobe bronchus in bronchoscopy [16]. The lesion was biopsied using a cryoprobe and histopathology showed a fibroepithelial polyp. The authors concluded that cryobiopsy is a useful technique for both enhanced sample size and a decrease in crush artifacts for the diagnosis of a fibroepithelial polyp of the bronchus [16].

The case we describe is original for three reasons. The first reason is that the first manifestation of the lesion was severe hemoptysis. According to a review of cases by Li et al. the most frequent first manifestation of bronchial fibroepithelial polyp was recurrent infection [5], while in a review by Casalini et al. the finding was incidental in the most of the cases [4]. The second reason is that, to our knowledge, this is the first time that bronchial fibroepithelial polyp occurs at CT as a thickening of a bronchus. Fibroepithelial polyps may be occasionally identified at CT as intraluminal, lobulated nodule with a blackberry-like appearance, especially when located into the main bronchi [4] and according to another review the most common radiological findings are obstruction and atelectasis [5]. The third reason is that in our case, it is the first time that bronchoscopic appearance of the polyp is bronchial mucosa invasion. Typical bronchoscopic appearance of fibroepithelial polyp that strongly supports the diagnosis is a whitish and glistening, firm, polypoid lesion with rounded or lobulated borders [4].

## Conclusions

Bronchial fibroepithelial polyps are rare clinical entities and are described as a handful of case reports. Medical doctors should always consider the possibility of these tumors in the differential diagnosis of hemoptysis. It is important to recognise fibroepithelial polyps because they may result in symptoms mimicking malignancy or infections and initial right diagnosis may prevent unnecessary surgical procedures. The current case report is unique, as to our knowledge for the first time in literature, bronchial fibroepithelial polyp is presented with severe hemoptysis as first manifestation, thickening of the posterior wall of the left main bronchus in CT of the chest and as mucosa invasion and not as whitish and glistening polypoid lesion with rounded or lobulated borders in bronchoscopic examination. 
